# Temporal Associations Between Daily Affect, Sleep, and Physical Activity in Family Caregivers of Older Adults

**DOI:** 10.1093/geroni/igaf122.4208

**Published:** 2025-12-31

**Authors:** Soojung Ahn, Dabin Hwang

**Affiliations:** Boston College William F. Connell School of Nursing, Chestnut Hill, Massachusetts, United States; Boston College William F. Connell School of Nursing, Chestnut Hill, Massachusetts, United States

## Abstract

Informal caregiving can be psychologically taxing and may disrupt caregivers’ ability to maintain health-promoting behaviors. Psychological distress is linked to sleep problems and reduced physical activity, yet their temporal relationships remain underexplored in caregiving contexts. This study examined within- and between-person associations among psychological distress, sleep, and physical activity in caregivers of older adults (≥65 years) with chronic illness. Thirty caregivers (mean age, 57 years [SD = 10]; female, 93.3%; white race: 76.7%) completed daily surveys for 7 days, reporting negative affect (e.g., depressed, bored, irritated, dissatisfied with self, worried, and nervous), perceived stress each evening, and sleep quality each morning. Concurrently, they wore an accelerometer (ActiGraph GT9X-Link) to capture sleep and physical activity. Multilevel models accounted for person-level covariates (age, general health status, depression, sleep disorder) and day-level covariates (weekday/weekend, lagged variables). Within-person analyses showed that on the days with higher levels of negative affect and stress, caregivers were more likely to experience shorter sleep duration (B [95% CI] = -1.08 [-2.10, -0.06]; -0.95 [-1.47, -0.43]) and to spend longer time being inactive the following day (3.08 [1.10, 5.06]; 1.41 [0.38, 2.44]). On the days following shorter or poorer-quality sleep, caregivers were more likely to spend longer time being inactive (-0.37 [-0.65, -0.08]; 44.20 [20.97, 67.44]). No statistically significant between-person differences were observed. These findings highlight the daily interdependence of psychological distress, sleep, and physical activity. Interventions should incorporate real-time strategies that address daily stressors, promote sleep hygiene, and encourage physical activity to support sustainable caregiver health and prevent long-term decline.

